# Supplementary material for: Early treatment of Favipiravir in COVID-19 patients without pneumonia: a multicentre, open-labelled, randomized control study
Source: Emerg Microbes Infect. 2022 Sep 21;11(1):2197–206. doi: 10.1080/22221751.2022.2117092 (PMC9518247; doi:10.1080/22221751.2022.2117092)
Supplement: Supplemental Material [file TEMI_A_2117092_SM8269.docx]

**Supplementary Methods**

**Sample size determination**

We assumed that there was a difference of roughly 4 days between FPV and control groups before viral-negative conversion. The median number of days to improvement of control patients’ respiratory status was assumed to be 9 days after symptomatic treatment administration (Day 10). The number of days to improvement for patients allocated to the FPV group was assumed to be 5 days after administration (Day 6). The survival function of the number of days to improvement was assumed to follow an exponential distribution. If α = 0.025 (one-sided), the power of detection is 80%, the allocation ratio of the control group to the FPV group is 1:2, and the probability of ineffective discontinuation of the control group is 3%, then a total of 96 subjects, consisting of 32 subjects in the control group and 64 subjects in the FPV group, will be required to establish the significance of treatment with favipiravir. The formula used for this sample size calculation is as shown below:

$$\text{N}\text{ }\text{=}\frac{\text{E}\text{ }\text{(α,}\text{ }\text{β,}\text{ }\text{ψ)}}{\text{ρ}_{\text{E}}\text{\{S}\left( \text{t} \right)\text{,}\text{ }\text{L}\left( \text{t} \right)\text{, R, T\}}}$$

**Primer design and qPCR**

RT-ddPCR specific primers and probes for SARS-CoV-2 RdRp were designed by the Thai National Institute of Health, and was sequenced as follows: RdRp forward primer 5’-CTCACCTTATGGGTTGGGATTATC-3’; RdRd reverse primer 5’-AGTGAGGCCATAATTCTAAGCATGT-3’; and RdRp probe FAM-3’-TAAATGTGATAGAGCCATGCC-5’-. 20 uL reactions consisted of: RT-PCR reagent [(One-Step RT ddPCR Advanced Kit for Probes, Bio-Rad Laboratories Inc., Hercules, CA), which included reverse transcriptase and DNA polymerase], 900 nM RdRp primers, 250 nM RdRp probe, and 5 uL of RNA sample. Droplet partitions (water-in-oil emulsion) were performed using a droplet generator (QX200TM AutoDG Droplet Digital PCR System, Bio-Rad Laboratories Inc., Hercules, CA, USA). The RdRp targeted gene was amplified by setting a thermal cycler (T-100TM, Bio-Rad Laboratories Inc., Hercules, CA, USA) according to the following conditions: 45℃ for 60 minutes, 95℃ for 10 minutes, 40 cycles of amplification at 95℃ for 15 seconds, 50℃ for 1 minute, and 98℃ for 10 minutes. Droplets were kept at 4℃ at least 10 minutes before reading FAM channels through the QX200TM Droplet Reader (Bio-Rad Laboratories Inc., Hercules, CA, USA).

**Supplementary Results**

Supplementary Table 1. NEWS Criteria.

| **Physiological Parameters** | **3** | **2** | **1** | **0** | **1** | **2** | **3** |
| --- | --- | --- | --- | --- | --- | --- | --- |
| *Respiration Rate* | ≤8 | - | 9-11 | 12-20 | - | 21-24 | ≥25 |
| *Oxygen Saturations* | ≤91 | 92-93 | 94-95 | ≥96 | - | - | - |
| *Any Supplemental Oxygen* | - | Yes | - | No | - | - | - |
| *Temperature* | ≤35.0 | - | 35.1-36.0 | 36.1-38.0 | 38.1-39.0 | ≥39.1 | - |
| *Systolic BP* | ≤90 | 91-100 | 101-110 | 111-219 | - | - | ≥220 |
| *Heart Rate* | ≤40 | - | 41-50 | 51-90 | 91-110 | 111-130 | ≥131 |
| *Level of Consciousness* | - | - | - | A | - | - | V, P, or U |

A: Alert, V: responds to verbal communication, P: responds to pain, U: unresponsive to stimuli.

Supplementary Table 2. Evaluation of criteria for clinical symptoms and findings.

| **Measured condition/symptom** | **Evaluation** | **Criteria** |
| --- | --- | --- |
| Patient's condition | 1 | Condition does not require hospitalization and does not interfere with ADL |
|  | 2 | Condition does not require hospitalization, but ADL is limited |
|  | 3 | Requires inpatient treatment, but no adjuvant oxygen therapy |
|  | 4 | Requires hospitalization and adjuvant oxygen therapy |
|  | 5 | Requires inpatient treatment and requires non-invasive ventilation therapy or a high-flow system |
|  | 6 | Requires hospitalization and invasive mechanical ventilation therapy or ECMO |
|  | 7 | Dead |
| Coughing,  sore throat, headache, muscle or joint pain, nasal congestion or discharge, chills or sweating, malaise or fatigue, diarrhoea | 3+ | Severe (symptoms are very worrisome and ADL is not possible) |
|  | 2+ | Moderate (symptoms are quite disturbing and interfere with ADL) |
|  | + | Mild (ADL is not compromised by symptoms) |
|  | - | None (no symptoms) |
|  | NA | Cannot be judged |
| Loss of taste, loss of smell, chest pain, dehydration, cyanosis, pleural effusion | + | Yes |
|  | - | No |
|  | NA | Cannot be judged |
| Thoracic rales | 2+ | Heard clearly |
|  | + | Faint |
|  | - | No |
|  | NA | Cannot be judged |
| Conscious state | U | Unresponsive (no response) |
|  | P | Responsive to pain (reacts to pain) |
|  | V | Responsive to voice (responds to voiceover) |
|  | A | Alert (clear) |

Supplementary Table 3. Clinical laboratory evaluations of SARS-CoV-2 infected patients 0-28 days after infection.

| **Clinical Laboratory Tests** | **Mean (SE)** | | | **P-value** |  |  |
| --- | --- | --- | --- | --- | --- | --- |
|  | **Case** | | **Control** |  |  |  |
| **(1) Complete Blood Count (CBC)** | | | | |  |  |
| - 1. **White Blood Cells (WBC) count** *(Reference range: female 4.5-8.0 x 10^3^/µL, male4.5-9.0 x 10^3^/µL)* | | | | |  |  |
| Day 0 (Screening days) | 5.24 (0.23) | | 5.15 (0.30) | 0.8210 |  |  |
| Day 4 | 5.14 (0.19) | | 6.45 (1.03) | 0.0939 |  |  |
| Day 7 | 7.17 (0.97) | | 6.15 (0.34) | 0.4680 |  |  |
| Day 10 | 5.84 (0.22) | | 7.00 (0.38) | 0.0051 |  |  |
| Day 28 | 6.45 (0.19) | | 6.68 (0.31) | 0.5047 |  |  |
| - 1. **Red Blood Cell (RBC) count** *(Reference range: female 3.7-5.0 x 10^6^/uL, male 4.0-5.7* | | | | |  |  |
| Day 0 (Screening days) | 4.86 (0.08) | | 4.88 (0.20) | 0.8760 |  |  |
| Day 4 | 4.80 (0.14) | | 5.05 (0.13) | 0.2409 |  |  |
| Day 7 | 4.89 (0.07) | | 5.08 (0.12) | 0.1721 |  |  |
| Day 10 | 4.79 (0.13) | | 5.06 (0.13) | 0.1638 |  |  |
| Day 28 | 4.56 (0.10) | | 4.67 (0.20) | 0.5813 |  |  |
| - 1. **Haemoglobin (Hb)** *(Reference range: female 11.0-14.0 g/dL, male 12.0-15.0 g/dL)* | | | | |  |  |
| Day 0 (Screening days) | 13.13 (0.32) | | 13.56 (0.30) | 0.3970 |  |  |
| Day 4 | 13.51 (0.29) | | 13.73 (0.31) | 0.6307 |  |  |
| Day 7 | 13.31 (0.26) | | 13.61 (0.30) | 0.4864 |  |  |
| Day 10 | 13.22 (0.30) | | 13.66 (0.29) | 0.3354 |  |  |
| Day 28 | 12.53 (0.24) | | 12.73 (0.26) | 0.5968 |  |  |
| - 1. **Haematocrit (Hct)** *(Reference range: female 35.0-41.0 %, male 38.0-48.0 %)* | | | | |  |  |
| Day 0 (Screening days) | 40.04 (0.80) | | 40.96 (0.82) | 0.4711 |  |  |
| Day 4 | 40.42 (0.79) | | 41.33 (0.84) | 0.4737 |  |  |
| Day 7 | 39.84 (0.71) | | 41.08 (0.77) | 0.2782 |  |  |
| Day 10 | 46.72 (7.39) | | 40.81 (0.76) | 0.5540 |  |  |
| Day 15 | 39.54 (0.97) | | 40.21 (1.71) | 0.7275 |  |  |
| Day 22 | 40.80 (0.97) | | 37.35 (0.35) | 0.1129 |  |  |
| Day 28 | 38.24 (0.63) | | 38.57 (0.66) | 0.7469 |  |  |
| **(2) Haematology Laboratory Normal Range (count.)** | | | | | |  |
| **2.1) Platelet** *(Reference range: 100-400 x 10^3^/uL)* | | | | | |  |
| Day 0 (Screening days) | | 243.25 (10.76) | 246.35 (10.58) | 0.8553 | |  |
| Day 4 | | 248.39 (12.75) | 237.07 (10.85) | 0.5725 | |  |
| Day 7 | | 275.28 (11.72) | 257.13 (11.81) | 0.3313 | |  |
| Day 10 | | 284.34 (11.59) | 277.05 (16.51) | 0.7140 | |  |
| Day 28 | | 293.67 (9.89) | 258.76 (12.82) | 0.0406 | |  |
| **(3) Differential WBC count** | | | | | |  |
| **3.1) Neutrophil** *(Reference range: female 35-75 %, male 36-70 %)* | | | | | |  |
| Day 0 (Screening days) | | 56.21 (1.82) | 54.87 (2.07) | 0.6534 | |  |
| Day 4 | | 52.40 (1.30) | 52.19 (1.41) | 0.9213 | |  |
| Day 7 | | 55.05 (1.46) | 52.06 (2.24) | 0.2520 | |  |
| Day 10 | | 58.32 (1.62) | 55.62 (2.19) | 0.3233 | |  |
| Day 28 | | 55.77 (0.82) | 55.34 (1.76) | 0.7990 | |  |
| **3.2) Lymphocyte** *(Reference range: female 20-59 %, male 23-57 %)* | | | | | |  |
| Day 0 (Screening days) | | 32.62 (1.45) | 35.17 (1.98) | 0.3062 | |  |
| Day 4 | | 36.63 (1.14) | 37.69 (1.47) | 0.5842 | |  |
| Day 7 | | 34.75 (1.24) | 38.56 (1.93) | 0.0893 | |  |
| Day 10 | | 31.20 (1.43) | 35.14 (1.96) | 0.1068 | |  |
| Day 28 | | 33.29 (0.83) | 33.89 (1.65) | 0.7171 | |  |
| **3.3) Monocyte** *(Reference range: 2-10 %)* | | | | | |  |
| Day 0 (Screening days) | | 8.61 (0.62) | 8.10 (0.45) | 0.5844 | |  |
| Day 4 | | 7.67 (0.38) | 6.93 (0.57) | 0.2709 | |  |
| Day 7 | | 7.26 (0.41) | 6.20 (0.43) | 0.1099 | |  |
| Day 10 | | 7.11 (0.25) | 5.72 (0.35) | <0.001 | |  |
| Day 28 | | 7.25 (0.22) | 6.48 (0.28) | 0.038 | |  |
| **3.4) Eosinophil** *(Reference range: 1-5 %)* | | | | | |  |
| Day 0 (Screening days) | | 1.86 (0.27) | 1.45 (0.36) | 0.3814 | |  |
| Day 4 | | 2.55 (0.26) | 2.61 (0.44) | 0.8947 | |  |
| Day 7 | | 2.34 (0.27) | 2.38 (0.37) | 0.9292 | |  |
| Day 10 | | 2.62 (0.24) | 2.97 (0.49) | 0.4746 | |  |
| Day 28 | | 3.44 (0.33) | 3.61 (0.45) | 0.7614 | |  |
| **3.5) Basophil** *(Reference range: 0-3 %)* | | | | | |  |
| Day 0 (Screening days) | | 0.27 (0.06) | 0.22 (0.07) | 0.6211 | |  |
| Day 4 | | 0.29 (0.06) | 0.28 (0.08) | 0.9191 | |  |
| Day 7 | | 0.39 (0.11) | 0.91 (0.66) | 0.2951 | |  |
| Day 10 | | 0.46 (0.07) | 0.38 (0.09) | 0.4644 | |  |
| Day 28 | | 0.54 (0.07) | 0.51 (0.90) | 0.8621 | |  |
| **(4) Clinical Chemistry** | | | | | | |
| **4.1) Albumin** *(Reference range: 3.5-5.0 g/dL)* | | | | | | |
| Day 0 (Screening days) | | 4.21 (0.07)  *(4.07, 4.35)* | 4.26 (0.06)  *(4.13, 4.38)* | 0.6233 | | |
| Day 4 | | 3.88 (0.09)  *(3.70, 4.06)* | 4.14 (0.05)  *(4.04, 4.25)* | 0.0500 | | |
| Day 7 | | 4.02 (0.04)  *(3.95, 4.09)* | 4.11 (0.04)  *(4.02, 4.20)* | 0.1578 | | |
| Day 10 | | 3.97 (0.04)  *(3.89, 4.05)* | 4.10 (0.06)  *(3.98, 4.23)* | 0.0633 | | |
| Day 28 | | 4.19 (0.04)  *(4.12, 4.27)* | 4.24 (0.05)  *(4.14, 4.34)* | 0.4728 | | |
| **4.2) BUN** *(Reference range: 6-20 mg/dL)* | | | | | | |
| Day 0 (Screening days) | | 10.32 (0.43) | 10.06 (0.44) | 0.7147 | | |
| Day 4 | | 10.50 (0.28) | 10.33 (0.35) | 0.7208 | | |
| Day 7 | | 11.21 (0.42) | 10.77 (0.50) | 0.5205 | | |
| Day 10 | | 10.85 (0.46) | 11.07 (0.59) | 0.7719 | | |
| Day 28 | | 11.97 (0.51) | 11.11 (0.69) | 0.3332 | | |
| **4.3) Alanine aminotransferase: ALT (SGPT)** *(Reference range: female 0-31 U/L, male 0-41 U/L)* | | | | | | |
| Day 0 (Screening days) | | 25.89 (2.36) | 27.48 (6.09) | 0.7694 | | |
| Day 4 | | 27.28 (2.31) | 24.76 (5.25) | 0.6104 | | |
| Day 7 | | 31.10 (2.61) | 24.63 (3.81) | 0.1601 | | |
| Day 10 | | 36.06 (3.41) | 24.55 (2.92) | 0.0258 | | |
| Day 28 | | 40.02 (4.73) | 31.63 (8.94) | 0.3823 | | |
| **4.3) Aspartate aminotransferase: AST (SGOT)** *(Reference range: female 0-31 U/L, male 0-37 U/L)* | | | | | | |
| Day 0 (Screening days) | | 28.33 (2.23) | 27.87 (2.63) | 0.9002 | | |
| Day 4 | | 26.70 (1.87) | 23.76 (1.87) | 0.3302 | | |
| Day 7 | | 28.65 (2.18) | 22.33 (1.89) | 0.0642 | | |
| Day 10 | | 28.22 (2.12) | 22.62 (1.18) | 0.0636 | | |
| Day 28 | | 26.64 (1.62) | 24.98 (3.02) | 0.5964 | | |
| **4.4)** **Alkaline phosphatase: ALP** *(Reference range: 35-129 U/L)* | | | | | | |
| Day 0 (Screening days) | | 63.49 (2.43) | 64.34 (7.46) | 0.8910 | | |
| Day 4 | | 66.92 (2.23) | 59.97 (3.05) | 0.0746 | | |
| Day 7 | | 72.01 (3.34) | 63.93 (3.36) | 0.1304 | | |
| Day 10 | | 69.42 (2.82) | 64.90 (4.25) | 0.3614 | | |
| Day 28 | | 68.22 (2.29) | 60.78 (3.74) | 0.0798 | | |
| **4.5) Calcium** *(Reference range: 8.6-10 mg/dL)* | | | | | | |
| Day 0 (Screening days) | | 9.20 (0.05)  *(9.10, 9.30)* | 9.29 (0.08)  *(9.12, 9.45)* | 0.3417 | | |
| Day 4 | | 9.14 (0.04)  *(9.05, 9.23)* | 9.32 (0.07)  *(9.17, 9.46)* | 0.0301 | | |
| Day 7 | | 9.29 (0.05)  *(9.18, 9.40)* | 9.38 (0.08)  *(9.21, 9.55)* | 0.3333 | | |
| Day 10 | | 9.60 (0.29)  *(9.02, 10.17)* | 9.53 (0.09)  *(9.35, 9.71)* | 0.8684 | | |
| Day 28 | | 9.33 (0.05)  *(9.22, 9.43)* | 11.14 (1.70)  *(7.66, 14.62)* | 0.1303 | | |
| **4.6) Phosphorus** *(Reference range: 2.7-4.5 mg/dL)* | | | | | | |
| Day 0 (Screening days) | | 3.44 (0.09) | 3.24 (0.09) | 0.1548 | | |
| Day 4 | | 3.55 (0.08) | 3.58 (0.08) | 0.8544 | | |
| Day 7 | | 3.58 (0.07) | 3.60 (0.09) | 0.9550 | | |
| Day 10 | | 4.62 (0.81) | 3.80 (0.09) | 0.4434 | | |
| Day 28 | | 3.43 (0.07) | 3.52 (0.23) | 0.6453 | | |
| **4.7) Potassium** *(Reference range: 3.5-5.1 mmol/L)* | | | | | | |
| Day 0 (Screening days) | | 3.83 (0.08) | 3.73 (0.04) | 0.3766 | | |
| Day 4 | | 3.91 (0.09) | 3.28 (0.05) | 0.5162 | | |
| Day 7 | | 4.53 (0.65) | 3.81 (0.06) | 0.4358 | | |
| Day 10 | | 3.93 (0.05) | 5.16 (1.28) | 0.2022 | | |
| Day 28 | | 5.96 (2.26) | 4.56 (0.84) | 0.6737 | | |
| **4.8) Sodium** *(Reference range: 136-145 mmol/L)* | | | | | | |
| Day 0 (Screening days) | | 136.24 (2.02) | 138.16 (0.50) | 0.5083 | | |
| Day 4 | | 137.97 (0.26) | 138.23 (0.32) | 0.5414 | | |
| Day 7 | | 136.77 (2.09) | 138.77 (0.39) | 0.5040 | | |
| Day 10 | | 137.21 (2.44) | 139.24 (0.49) | 0.5385 | | |
| Day 28 | | 136.51 (2.68) | 128.92 (6.57) | 0.1786 | | |
| **4.9) Chloride** *(Reference range: 98-107 mmol/L)* | | | | | | |
| Day 0 (Screening days) | | 101.92 (1.65) | 102.94 (0.39) | 0.6628 | | |
| Day 4 | | 103.25 (0.29) | 99.17 (3.25) | 0.0965 | | |
| Day 7 | | 103.57 (0.34) | 104.23 (1.14) | 0.4791 | | |
| Day 10 | | 102.54 (1.84) | 102.93 (0.41) | 0.8767 | | |
| Day 28 | | 103.3 (0.27) | 100.91 (3.48) | 0.3294 | | |
| **4.10) Uric acid** *(Reference range: female 2.4-5.7 mg/dL, male 3.4-7.0 mg/dL)* | | | | | | |
| Day 0 (Screening days) | | 5.03 (0.18) | 5.30 (0.29) | 0.4071 | | |
| Day 4 | | 8.68 (0.18) | 5.10 (0.22) | <0.001 | | |
| Day 7 | | 9.08 (0.25) | 5.26 (0.31) | <0.001 | | |
| Day 10 | | 8.93 (0.29) | 5.74 (0.35) | <0.001 | | |
| Day 28 | | 5.58 (0.18) | 5.92 (0.46) | 0.431 | | |
| **(5) Clinical Immunology Laboratory Normal Range (Cont.)** | | | | | | |
| **5.1) C-Reactive Protein (CRP)** *(Reference range: 0-1 ug/dL)* | | | | | | |
| Day 0 (Screening days) | | 0.77 (0.14) | 1.26 (0.47) | 0.2080 | | |
| Day 4 | | 0.85 (0.19) | 0.513 (0.19) | 0.2714 | | |
| Day 7 | | 0.79 (0.16) | 0.81 (0.23) | 0.9576 | | |
| Day 10 | | 0.52 (0.11) | 0.77 (0.26) | 0.3099 | | |
| Day 28 | | 0.55 (0.13) | 0.75 (0.20) | 0.3880 | | |
| **5.2) Procalcitonin (PCT)** *(Reference range: 0- 0.5 ng/dL)* | | | | | | |
| Day 0 (Screening days) | | 0.46 (0.12) | 0.56 (0.31) | 0.7158 | | |
| Day 4 | | 0.47 (0.15) | 0.61 (0.31) | 0.6245 | | |
| Day 7 | | 0.52 (0.19) | 0.33 (0.16) | 0.5379 | | |
| Day 10 | | 0.34 (0.14) | 0.36 (0.18) | 0.9490 | | |
| Day 28 | | 0.59 (0.16) | 0.49 (0.22) | 0.7170 | | |
| **(6) Cardiovascular Lab** | | | | | | |
| **6.1) QT interval** *(Reference range: female < 470 ms, male < 450 ms)* | | | | | | |
| Day 0 (Screening days) | | 378.32 (5.91) | 379.63 (3.79) | 0.8834 | | |
| Day 14 | | 427.45 (5.81) | 401.68 (6.63) | <0.001 | | |
| Day 28 | | 392.60 (3.19) | 378.72 (4.93) | <0.001 | | |
| **6.2) PR interval** *(Reference range: 120-200 ms)* | | | | | | |
| Day 0 (Screening days) | | 156.66 (8.10) | 144.07 (3.44) | 0.2904 | | |
| Day 14 | | 153.49 (4.55) | 147.89 (5.38) | 0.4612 | | |
| Day 28 | | 152.68 (2.81) | 149.17 (4.79) | 0.5042 | | |

Supplementary Table 4. Prevalence of adverse events by group.

| **Adverse Event** | **Favipiravir (n=62) (%)** | **Control (n=31) (%)** |
| --- | --- | --- |
| Any AE (e.g., unspecified fever, upper respiratory tract infection, acute lymphadenitis right cervical, steroid acne, etc.) | 10 (16.1) | 2 (6.5) |
| Constipation | 2 (3.2) | - |
| Diarrhoea | 1 (1.6) | - |
| Dizziness | 1 (1.6) | - |
| Dyspepsia | 1 (1.6) | - |
| Insomnia | 2 (3.2) | - |
| Rash | 3 (4.8) | - |
| Hypertension | - | 1 (3.2) |
| Hypertriglyceridemia | 2 (3.2) | - |
| Hyperuricemia | 11 (17.7) | 1 (3.2) |
| Hypokalaemia | 6 (9.7) | 7 (22.6) |
| Hypoleukaemia | 1 (1.6) | - |
| Increased CPK | - | 1 (3.2) |
| Increased SGPT | 1 (1.6) | - |
| Anaemia | 2 (3.2) | - |
| Leukopenia | 1 (1.6) | - |
| Hyperglycaemia | 2 (3.2) | - |


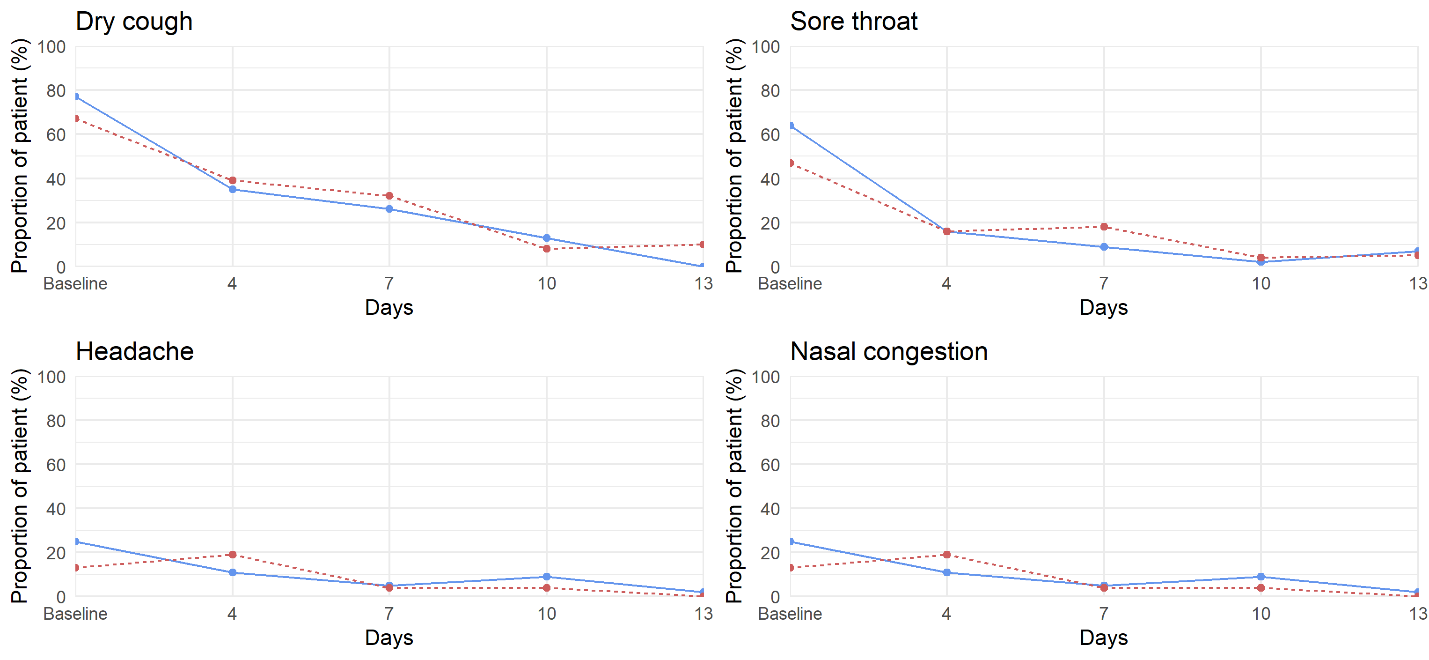

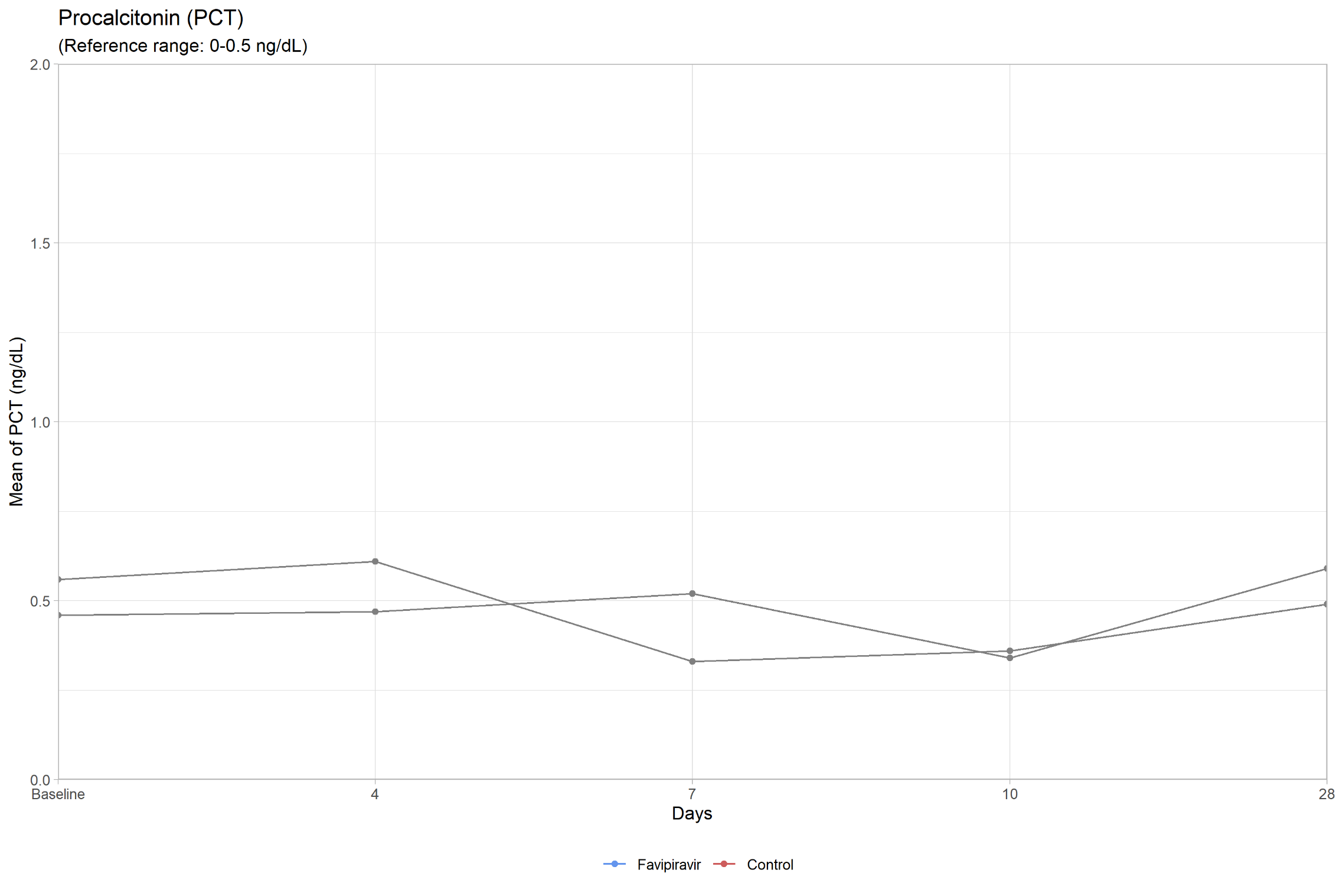


Supplementary Figure 1. Changes in clinical symptoms (dry cough, sore throat, headache, and nasal congestion) over 0-13 days. Line graphs illustrate the proportion of patients who had clinical symptoms each day after enrolment.

**
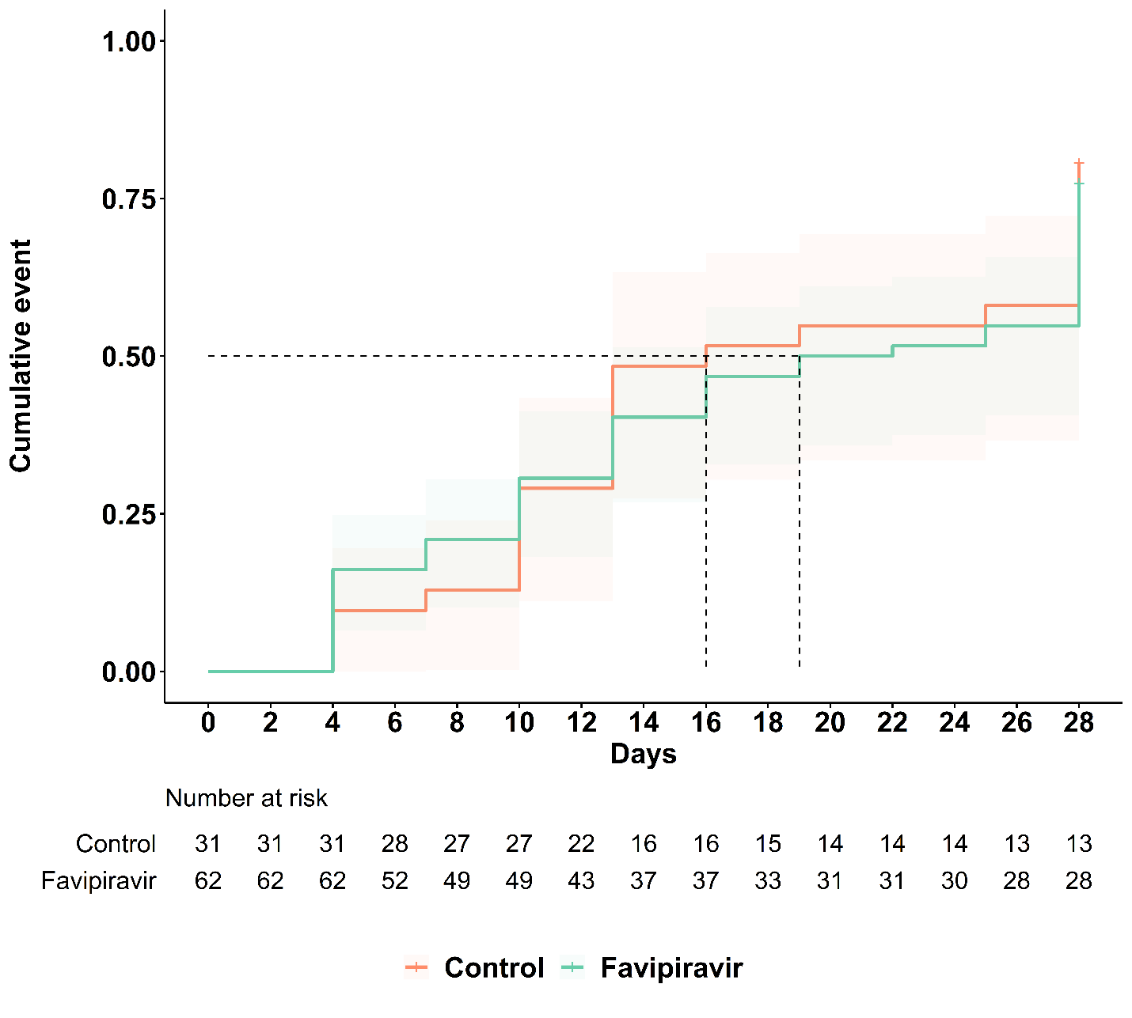
**

Supplementary Figure 2. Time to undetectable virus over 28 days. The Kaplan-Meier curve illustrates the cumulative proportion of patients who had undetected viral titres over 28 days. The median time to undetected virus was 19 days vs 16 days (interquartile range (IQR) of 10-28 days for both) for FPV and control arms respectively (adjusted hazard ratio (aHR) 0.96, 95% CI: 0.58-1.58, *P* = 0.871).


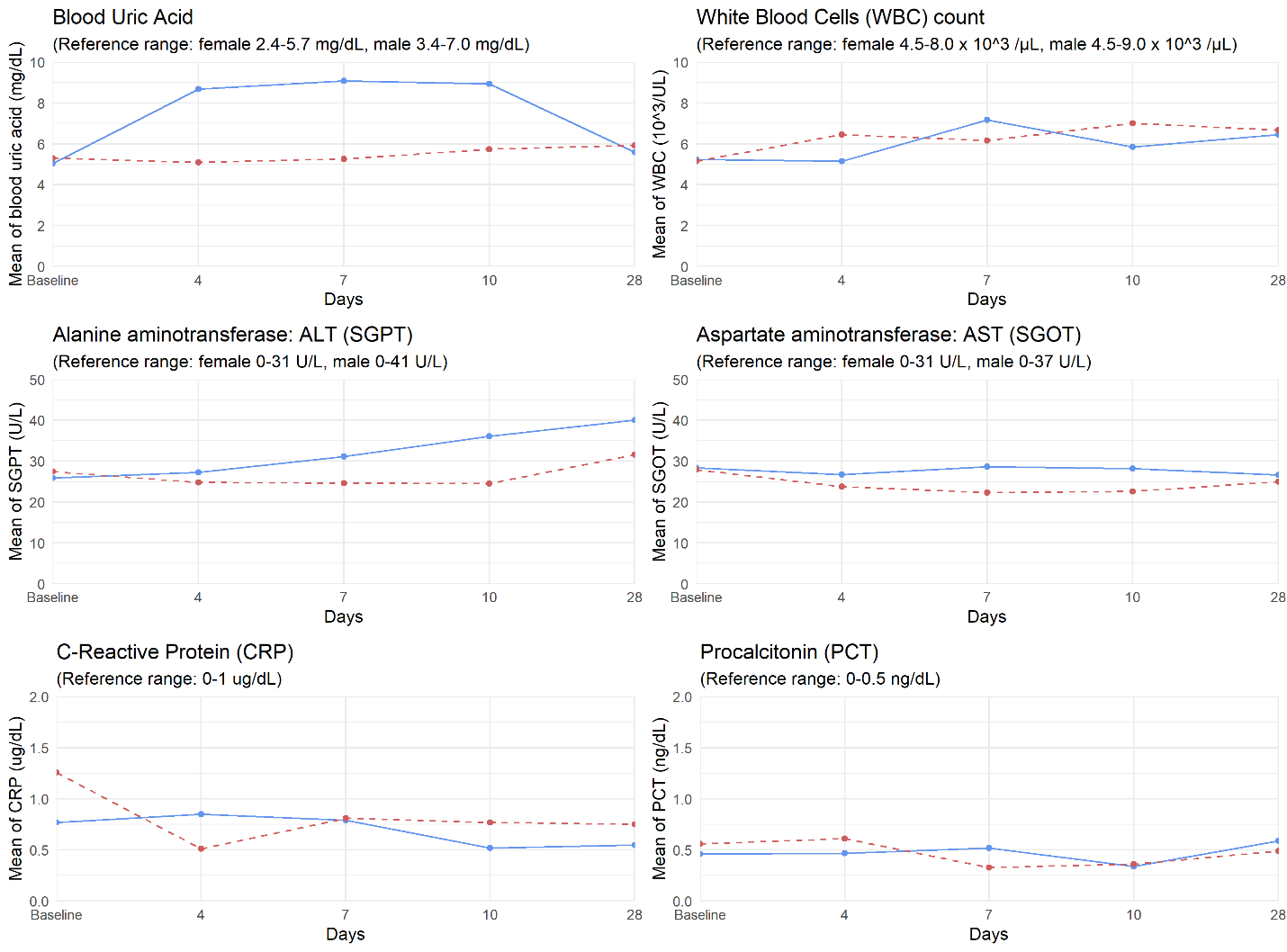

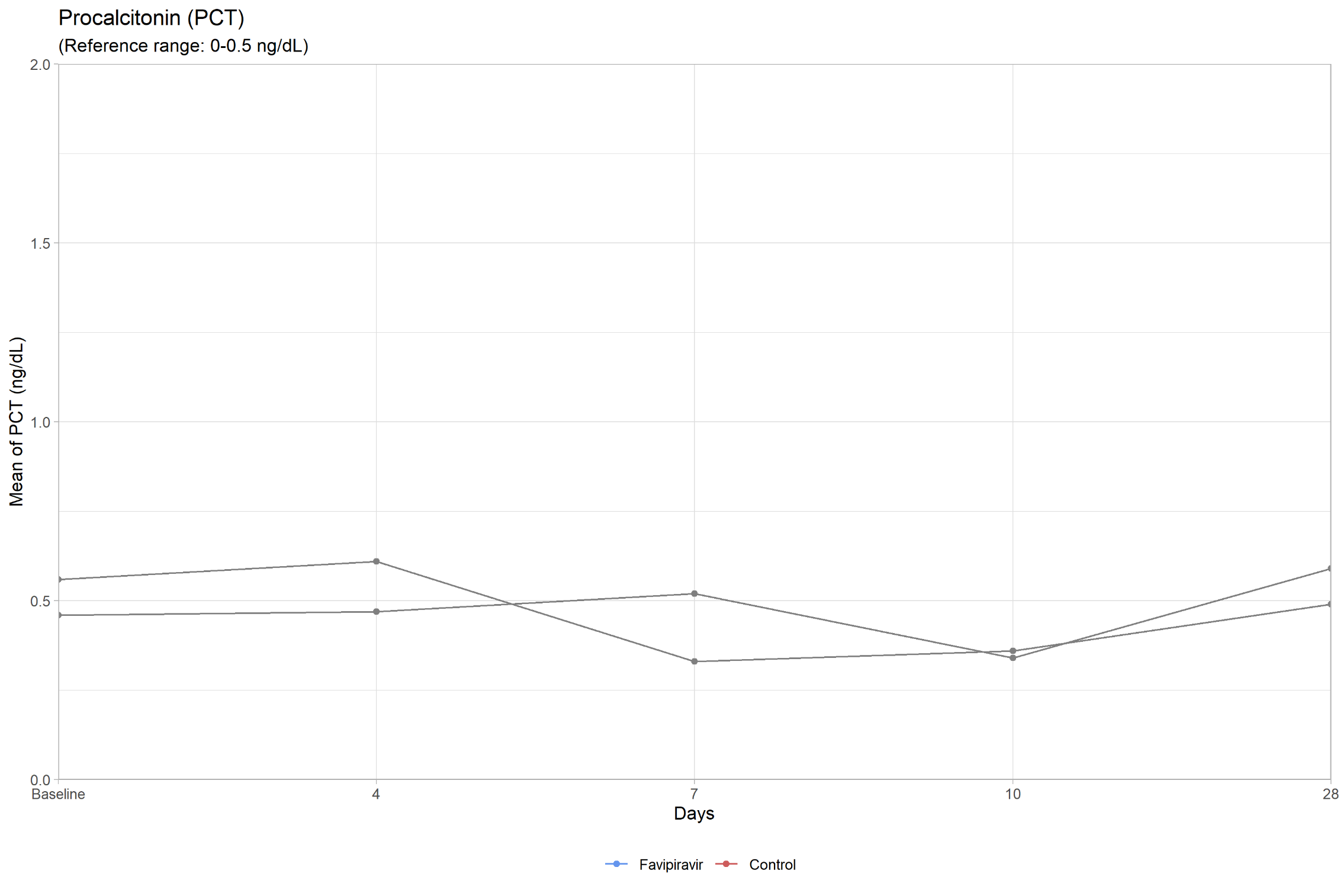


Supplementary Figure 3. Clinical laboratory evaluations of blood uric acid, white blood cell (WBC) count, alanine aminotransferase (ALT), aspartate aminotransferase (AST), C-reactive protein (CRP), and procalcitonin (PCT). Line graphs illustrate the proportion of patients who had clinical symptoms from days 0-28.
